# Concepts for migration-sensitive health monitoring

**DOI:** 10.25646/6075

**Published:** 2019-09-18

**Authors:** Maria Schumann, Katja Kajikhina, Antonino Polizzi, Navina Sarma, Jens Hoebel, Marleen Bug, Susanne Bartig, Thomas Lampert, Claudia Santos-Hövener

**Affiliations:** Robert Koch Institute, Berlin Department of Epidemiology and Health Monitoring

**Keywords:** MIGRATION, HEALTH MONITORING, ACCULTURATION, DISCRIMINATION, SUBJECTIVE SOCIAL STATUS

## Abstract

According to microcensus data, nearly one quarter of the German population has a migration background. This means that either themselves or at least one parent was born without German citizenship. Based on the currently available data and due to the underrepresentation of specific population groups, representative findings on the health of the total population residing in Germany are only possible to a limited degree. Against this backdrop, the Robert Koch Institute initiated the Improving Health Monitoring in Migrant Populations (IMIRA) project. The project aims to establish a migration-sensitive health monitoring system and to better represent people with a migration background in health surveys conducted by the Robert Koch Institute. In this context it is crucial to review and further develop relevant migration-sensitive concepts and appropriate surveying instruments. To achieve this, the concepts of acculturation, discrimination, religion and subjective social status were selected. This article theoretically embeds these concepts. Furthermore, we describe their application in epidemiology as well as provide a proposal on how to measure and operationalise these concepts. Moreover, recommendations for action are provided regarding the potential application of these concepts in health monitoring at the Robert Koch Institute.

## 1. Introduction

Since its founding, the Federal Republic of Germany has been an immigration country [1]. Based on microcensus data, in 2017, around 19.3 million people living in Germany have a migration background [2]. This means that either themselves or at least one parent was born without German citizenship. Due to the heterogeneity of this population group, general statements on the health of people with a migration background should be avoided. However, numerous studies that applied a more nuanced analysis of living situations show that disease risks and health resources can vary depending on a set of factors such as region of origin, reasons for migration as well as people’s experiences before, during and after migration.

In studies on health status, health behaviour, the utilisation of medical services, health promotion and prevention services in the general population, people who are included in the category “migration background” are so far mostly underrepresented. In spite of this population group comprising around one quarter of the total population, validated data is nonetheless lacking [3, 4]. Surveys on migration and health tend to merely consider whether a person has a migration background (yes/no), an approach that does not adequately reflect the heterogeneity of people living in Germany [5].

Against this backdrop, the Robert Koch Institute (RKI) initiated the Improving Health Monitoring in Migrant Populations (IMIRA) project. The objective is to establish migration-sensitive health monitoring, better reach people with a migration background and include them in Robert Koch Institute health surveys on a longitudinal basis [5-7]. One of the IMIRA sub-projects aims to adapt and further develop concepts (sub-project 2) of migration-sensitive health monitoring. The objective is to identify migration-sensitive concepts that would reach beyond the minimum indicator set currently applied to operationalise migration background as developed by Schenk et al. [8]. Beyond citizenship and country of birth, the indicator set contains parent’s country of birth, year of immigration to Germany, German as mother tongue (yes/no), self-assessed German language proficiency (very good, good, average, poor, very poor) and residency status (temporary, permanent, German citizenship) [8]. Additionally, research into the current scientific literature led to the identification of further relevant concepts in the field of migration and health. Among these were the concepts of acculturation, discrimination, religion as well as subjective social status. The aim is to better illustrate important aspects of the different living situations of the population living in Germany and to better explain health inequalities.

The concepts identified, their operationalisation and measurement as well as the recommendations to establish migration-sensitive health monitoring at the RKI are based on an ideal approach that, potentially, will be applied in the future. This would provide opportunities to use the chosen items on migration history (including country of birth, citizenship, duration of stay) and discrimination experiences as well as sense of belonging, religion and subjective social status within future health surveys by the RKI. Already, the relevant dimensions of the concepts identified have largely become part of the core indicator set used to analyse the health of people with a migration background in health reporting (see the article Health reporting on people with a migration background – Selection and definition of (core)indicators in this issue). Besides migration background, these include the country of birth, German language proficiency, social networks as well as discrimination experiences. Duration of stay, residency status, reasons for migrating, feeling of belonging and religion are all part of the extended indicator set.

This article presents the selected concepts in more detail. It provides a theoretical basis, an overview of the concept’s application in epidemiology thus far as well as a proposal for measurement and operationalisation. Surveying instruments were selected based on both previous research findings and ethical principles developed by civic organisations. Moreover, they were cognitively tested and subsequently optimised in the six languages (Arabic, German, Italian, Croatian, Polish and Turkish) which correspond to the overall population for the upcoming Health and Nutrition Survey in Germany (gern survey), a joint study by the Robert Koch Institute and the Max Rubner Institute. Cognitive testing included interviews of participants of different age, sex and educational levels who were native speakers of the six languages mentioned. After completing the written questionnaire, which contained all the items to be tested, respondents were asked about their feelings, understanding of the questions and how to answer them using a standardised interview guide. The partial transcripts were then qualitatively analysed using the Constant Comparative Method [9]. Based on these findings the questionnaire was then adapted where necessary. The article closes with recommendations on those aspects that should be considered when using the presented concepts in epidemiological research.

## 2. Acculturation

Originally, the concept of acculturation was developed in British and North American anthropology as an attempt to describe the process of cultural changes in people as a result of the contact between people from different cultures during the colonial era at the end of the 19th century [10, 11]. Today, the concept has been applied in numerous further disciplines, such as medicine, psychology, public health and epidemiology [12, 13]. Numerous studies on the connection between acculturation and health have been conducted [14-20]. The results show great inconsistencies regarding the direction and strength of effects shown [21]. General conclusions regarding the interdependencies between acculturation and health are therefore not possible [22].

In principle, there are three distinguishable theoretical approaches. One-dimensional approaches describe acculturation as a linear continuum stretching from not acculturated to acculturated. These approaches view acculturation as a process of transition from a person’s original culture to the new one [23]. Two-dimensional approaches of acculturation in turn understand acculturation as a process taking place on two different planes. People with a migration background thereby integrate elements of the new culture as well as discard elements of their original culture [24, 25]. Multidimensional approaches take this one step further and consider additional dimensions that are surveyed independently [26]. All approaches have in common that they attempt to calculate an acculturation score and provide a statement on a person’s degree of acculturation.

As part of IMIRA sub-project 2, a systematic review of the literature was conducted to (1) provide an overview of the degree to which the concept of acculturation has already been used in epidemiologic research on the health of people with a migration background; (2) identify the different options of operationalising and measuring the concept of acculturation; and (3) develop recommendations to survey the concept of acculturation in the context of health surveys in Germany (original papers with quantitative approaches in German and English; search strategy applying a combination of terms such as acculturation, migration, method, health; specialised data bases MEDLINE, SCOPUS and Science Direct; further information and criteria can be found at [27]).

### 2.1 Operationalising and measuring acculturation

The majority of the studies identified were conducted in the US, yet a few also in Europe. These mainly analyse the links between the acculturation of diverse groups of people with a migration background and/or who have different ethnic backgrounds with regard to different health outcomes. Due to significant inconsistencies related to the definition, operationalisation and measurement of the concept in the studies considered, no general statements on the impact of processes of acculturation on the health of people with a migration background are possible [16, 26, 28].

For example, these studies have used a diverse set of proxy variables and acculturation scales. These are used to collect data on migration history, language, (ethnic) background and a person’s social environment. Based on these dimensions, an acculturation score is then usually calculated that aims to provide statements on a person’s degree of acculturation. In this context, however, it remains unclear how the multiple dimensions relate to each other and whether changes in one dimension (such as greater language proficiency) also lead to changes in other dimensions (such as stronger social networks) [29, 30].

Due to the numerous gaps regarding the theoretical establishment of the concept of acculturation, the inconsistencies related to its operationalisation and measurement as well as a critical discussion of an approach that generalises and categorises people as members of a ‘culture’ (based on ascribed and often ethicising traits), we have decided not to introduce the concept of acculturation as such or to calculate an acculturation score in the health studies of the Robert Koch Institute. Nonetheless, our review identified four thematic dimensions within the scales measuring acculturation in the literature. Based on those, proxies and items were identified that we intend to survey within future migration-sensitive health monitoring. These would be a person’s migration history, their language skills (German and native language), subjective feeling of belonging and social support. These relevant factors need to be taken into consideration when explaining differences in health/health outcomes of people with a migration background.

### 2.2 Operationalisation and measurement of relevant dimensions

#### Migration history

For migration history, the variables within the minimum indicator set by Schenk et al. [8] were revised and supplemented. Beyond country of birth and citizenship, parents’ country of birth as well as data on the year of immigration to Germany would be collected. Collecting data on parent citizenship is also recommended. Furthermore, this data would allow researchers to calculate how long a person has been in the country of residence, the number of years that have passed since migration and their age at immigration. Regarding residency status, we would basically apply the categories developed by the Federal Office for Migration and Refugees (BAMF), yet adapt them where necessary [31]. Cognitive testing indicated that translated questionnaires should also provide the answer categories in German. Frequently, respondents learn the German terminology of residency regulations (for example Erlaubnis zum Daueraufenthalt in der Europäischen Union, Aufenthaltsgestattung or Duldung) after arriving in Germany and therefore appear to use them mostly in German. Often, they either do not know or there is no equivalent term in their native language. A new aspect would also be to collect data on the reasons for migration (motivation). The motivating reasons for migration we propose are also based on the categories of surveys by BAMF (for employment, training, according to international law, humanitarian or political refugees, family, late repatriates or other reasons) [32]. Following cognitive testing, these were supplemented by further frequently mentioned reasons such as the answer ‘to have a better future’.

#### Language

Language surveying was also further developed. The minimum indicator set [8] only collected data on German as a native language (yes/no) as well as a person’s self-assessed German language skills (very good, good, average, poor, very poor). We now recommend a four-step approach. In a first step, data on a person’s native language would be collected by asking ‘Which language is your native language?’ followed by a list of ten languages plus a box to enter any other language. The ten languages selected would cover the most frequently spoken languages in the overall population including German. These would include Arabic, Italian, Croatian, Polish and Turkish. In a second step, like in the minimum indicator set, respondents would be asked to self-assess their language proficiency. This would initially aim to collect data on German proficiency based on Brand et al. [15] by using the item ‘How good do you think your German is?’ on a five item answer scale (very good, good, average, poor, very poor). The third step would consist of surveying a person’s native language (except German) with the item ‘How well do you speak your native language (except German)?’ followed by the same five possible answers. Moreover, the language used during the interview (or in the questionnaire) would be established as a proxy variable.

#### Feeling of belonging

From our perspective, surveying the subjective feeling of belonging in German society should be conducted analogous to the migration sample of the Institute for Employment Research (IAB), the Socio-Economic Panel (SOEP) of the German Institute for Economic Research (DIW Berlin) as well as the representative survey Ausgewählte Migrantengruppen in Deutschland (RAM 2015) by the Federal Office for Migration and Refugees (BAMF). The item ‘How strongly do you feel you are a part of German society?’ would be followed by a five item Likert scale (very strongly, strongly, partly, barely, not at all). The feeling of belonging to the country of origin would also be implemented by using a corresponding answer scale based on the IAB-SOEP migration sample template and the RAM of the BAMF: ‘And how strongly do you feel you are a part of the society of your country of origin (i.e. the country in which you or your parents were born)?’ It is thereby important to recognise that surveying a subjective feeling of belonging in the context of health is relevant not only for people with a migration background. The subjective feeling of belonging also influences the health of other population groups, which is why surveying this feeling should not be limited to the migration sample.

#### Social support

A person’s social environment emerged as the fourth global dimension from the review of the literature on the concept of acculturation. This included proxies as well as items within acculturation scales that survey for example the origin of friends or people in the neighbourhood, or contact with family members in the country of origin, but also knowledge of political and social questions, dietary habits and other items that aim to operationalise belonging to a culture. These questions on the social environment operationalised in this manner are only posed to people with a migration background. The reason behind these questions is an idea of fixed and generalisable traits and social interactions that are thought to define or nullify a supposed belonging to a category ‘culture’ which itself is based on unclear assumptions and attributions.

Against this backdrop, we recommend mapping the social environment as part of an approach for migration-sensitive health monitoring at the Robert Koch Institute based on social support and using none of the above-mentioned ‘acculturation’ items. These would – as done in previous health surveys of the RKI – be surveyed according to the Oslo 3 Social Support Scale. The scale is an instrument frequently applied in Europe to measure perceived social support [33, 34]. Participants thereby answer three questions with a four item response format on the number of people they would consider themselves close to or people that they can rely on, how many other people are in their lives and the availability of neighbourhood assistance.

## 3. Discrimination

Interpersonal and structural discrimination play a crucial role in physical and mental health. People can suffer from racist discrimination independently of whether they have a migration background according to the the current microcensus definition or not (for example on grounds of origin, appearance, accent, name). Surveying data on discrimination in the context of health monitoring at the RKI potentially provides a basis to analyse perceived discrimination and the health of those affected. At the RKI, subjectively felt discrimination has been surveyed once in the German Health Interview and Examination Survey for Children and Adolescents (KiGGS). However, it became clear that, concerning the methodological and ethical recommendations to collect data on discrimination, the instruments applied would have to be optimised in terms of the dimensions surveyed and the terminology used [35, 36].

An analysis of current research regarding the link between discrimination and health was carried out (reviews and original studies, search strategy using combined MeSH terms such as discrimination, racism, health, mental health, healthcare, gender, socioeconomic status, MEDLINE database). In addition, different survey instruments were compared and discussed that have already been used in repeated representative surveys in Germany, including the RKI’s KiGGS study and the surveys by the Socio-Economic Panel, by the Berlin Institute for Integration and Migration Research, as well as the Federal Government’s anti-discrimination agency. Furthermore, a summary of the recommendations on methodological and ethical principles to survey ethnicity and anti-discrimination data that have been developed for German-speaking countries by civil society institutions, the federal anti-discrimination agency and research institutions was developed [35-37].

### 3.1 Operationalising and measuring subjectively perceived discrimination

The majority of studies were published in North America, with a smaller number coming from European countries. They show a clear correlation between discrimination and health when outcomes such as mental health, overall/physical health, health behaviour and use of healthcare and prevention services are considered. A variety of scales, individual items and instruments are used to operationalise perceived discrimination as subjectively worse or less favourable treatment in everyday life, in relevant areas of life (offices, shops) or disadvantage in structural areas such as the housing market or in professional life. Studies survey the possible grounds for discrimination as well as the frequency of experienced discrimination. A large number of publications explicitly examines the connection between racial discrimination and health [38, 39].

There are important methodological and ethical aspects to consider when collecting anti-discrimination data. Information needs to be provided voluntarily, external ascriptions have to be avoided and the relevant groups should participate in developing the content and instruments, data collection and analysis. Ensuring an intersectional perspective, i.e. collecting data on all dimensions of discrimination without limiting the perspective to single factors such as migration background or country of origin is also essential [35].

On this basis, we have developed a proposal for a three stage approach. Initially, we would survey the subjectively perceived frequency of interpersonal discrimination experiences in people’s everyday lives by applying the 5-item everyday discrimination scale [40, 41]. The scale asks ‘In your day-to-day life how often have any of the following things happened to you?’ and offers the following situations as answers: ‘You receive poorer service than other people at restaurants or stores.’, ‘You are treated with less respect’, ‘People act as if they think you are not smart.’, ‘People act as if they are afraid of you.’, ‘You are threatened/harassed’. This 5-item variant of the scale has been widely used in English-speaking countries and validated for numerous population groups. Moreover, the scale has already been adapted and applied to some concrete settings, for example, to investigate discrimination in a medical setting [42]. The frequency of experiences of discrimination is surveyed with a 5-item Likert scale (very often, often, sometimes, rarely, never).

The following question asks about the possible reasons for the reported experiences also with regard to possible ascriptions and allows multiple answers: ‘What do you think was the main reason for these experiences? (Multiple answers possible. Please also consider how others possibly see you)’. We also formulate answers according to the established item set of the everyday discrimination scale [40, 41] as well as based on the six categories of the general equal treatment act: ‘sex’, ‘origin, accent, language, appearance, name’, ‘religion’, ‘chronic disease, impairment’, ‘sexual orientation’, ‘age’ and supplement these with ‘weight’, socioeconomic status (‘education, income’) and ‘unemployment’.

We would also expand the instrument to include questions on the frequency of discrimination experiences in the health and care sector and in contact with authorities: ‘How often have you been treated unfairly or worse than other people in the following situations? In the health or care sector (for example during a doctor’s appointment, at hospital, assisted living, care facility), during contacts with public offices or authorities (e.g. registration offices, immigration offices, job centres, police)’. The frequency of experienced discrimination will again be surveyed by using the Likert scale. These questions aim to collect data in the fields particularly relevant to health monitoring. Potentially, it will enable the identification of subjectively perceived barriers to access healthcare and health services due to discrimination. Yet discrimination also has public health relevance in people’s dealings with administrative offices, institutions and public facilities, for example when applying for health services. These two structural areas were selected following the evaluation of the cognitive pre-tests conducted with relevant groups of persons.

## 4. Religion

The term religion is defined as the belief in a higher power resulting from belonging to a religious creed such as Christianity, Islam, Judaism, Buddhism or Hinduism [43]. During recent years, a series of empirical studies has investigated the influence of religion on physical and mental health as well as mortality [44-46].

According to the proposal for health monitoring at the RKI, the initial question will address the belonging to one of the religious creeds. Similar to the study Deutschland postmigrantisch by the Forschungsgruppe Junge Islambezogene Themen in Deutschland (JUNITED) at the Berlin Institute for Integration and Migration Research (BIM) at the Humboldt University of Berlin, one question would have to be: ‘What is your religion?’ The possible answers would be the five major world religions (Christianity, Islam, Judaism, Hinduism, Buddhism), plus the options ‘any other religion’, ‘no religion’, as well as ‘I prefer not to answer this question’. Originally, our concept foresaw surveying participation in community life as a further social resource. However, the results of the cognitive pre-tests showed that the translations of the concept of community life give rise to very different associations. As a result, this question was not included in the set of recommended questions. The question of the connection between religion, well-being and life satisfaction is, however, of particular health relevance. We are therefore considering including the question: ‘How important is religion for your everyday well-being and life satisfaction?’ followed by a four item answer scale (very important, slightly important, not so important, not important at all). The survey conducted by the Socio-Economic Panel on religion as a resource of social cohesion already applied this question in this manner [47].

## 5. Subjective social status

Numerous empirical studies consistently show that people with a low socioeconomic status (SES) face an increased risk of developing disease and dying at a younger age [48-51]. Conceptually, SES is understood as a multidimensional construct and classically measured based on the three ‘objective’ indicators of education, occupation and income [52, 53]. SES measurement is therefore based on factors that significantly influence the position of people at the ‘top and bottom’ of society [54]. Using objective SES indicators attributes a status position to people. However, this attribution is not always congruent with how people assess their own status. To account for this subjective perspective, health research is increasingly supplementing objective SES indicators with subjective status indicators [55-57]. Subjective social status (SSS) indicates where people see themselves on the social ladder and the status group they feel they belong to.

Research in recent years indicates that a lower SSS, independently of objective SES, is associated with poorer physical and mental health [55, 56, 58-60]. Such an association between SSS and health has also been observed among people with a migration background [61]. The association is not only evident in cross-sectional studies. Observations from longitudinal studies and experimental studies also indicate that SSS has an independent effect on health and disease risks [62-65]. It is assumed that a low SSS reflects perceived social disadvantages and relative deprivation, which in turn cause feelings of injustice, anger, inferiority or shame [66-68]. Permanent or recurring feelings of this kind could lead to a chronic state of stress and thus to an increased risk of disease for people with low SSS [68]. Partly, however, SSS could also express aspects of socioeconomic conditions that are not covered by the three classic SES indicators such as wealth, over-indebtedness or social security.

Often it is difficult to assess people’s SES priorto migration, i.e. in their country of origin. Generally, it is difficult to translate their socioeconomic conditions in their countries of origin to the context of their country of residence, such as educational attainment or the status their occupation grants them. In these cases, SSS could provide an adequate and, for research, pragmatic solution. It should be noted, however, that SSS cannot fully substitute objective SES indicators, especially as the effects of objective and subjectively perceived living conditions on health would then become indistinguishable.

### 5.1 Measuring SSS in the country of residence (Germany)

**Figure 1:**
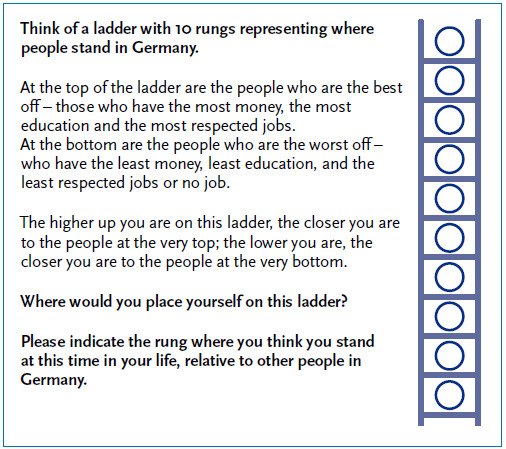
German version of a MacArthur Scale to assess the subjective social status of adults Source: Hoebel et al. 2015 [57]

In health research and epidemiology, the MacArthur Scale of Subjective Social Status has internationally established as the standard instrument to measure SSS [55, 56]. The instrument includes a ladder with ten rungs representing social structure. Interviewees are asked to mark where they would position themselves on this social ladder. The texts contained in the MacArthur Scale thereby explicitly reference the classical socioeconomic factors of education, occupation and income. Relative self-assessment generally takes place with regard to the society of the country in which respondents live. The MacArthur Scale can thus be applied to people with and without a migration background. Figure 1 shows the German version of the MacArthur Scale which has been used in RKI surveys to collect data on SSS in Germany and which has already been tested for construct validity in the general population [57].

### 5.2 Measuring hypothetical SSS in the country of origin

**Figure 2:**
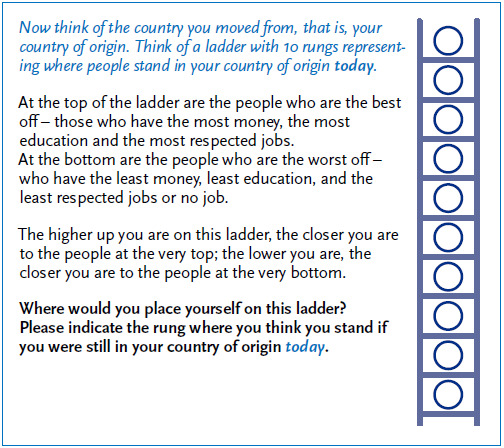
Adapted version of the MacArthur Scale to assess the subjective assessment of the hypothetical social status in the country of origin for people with a migration background^*^ Source: Own figure
*The text is a translation based on the German-language version of the questionnaire item. It has not yet been validated for use in questionnaire surveys and is only included in this manuscript for reasons of readability.

One US study [69] applied a MacArthur Scale-based questionnaire to collect data on subjective social status. At the point of interview, people who have migrated were asked to assess what their current social position in their country of origin would be if they had continued to live there [70]. Figure 2 shows the adapted version of the instrument in German, which has already been applied in an RKI feasibility study. While country of residence SSS data is collected independently by health studies and can be used as an (in)dependent variable, collecting data on the hypothetical, country of origin SSS of migrants is generally not done separately, but in combination with measuring country of residence SSS.

One question that can be addressed by combining both items is, for example, whether a discrepancy between the hypothetical SSS in the country of origin and the SSS in the country of residence has a health impact. For some immigrant population groups in the US, it has been observed that on average the health of people who consider their status in the country of residence to be lower than their hypothetical status in their country of origin is poorer [70, 71]. The control group was comprised of migrants not perceiving such a status discrepancy. However, this association played out differently depending on the country of origin [70]. So far, the mechanisms underlying these results have not been sufficiently clarified. Possibly, however, perceived downward social mobility due to migration or counterfactual thinking (‘Had I never migrated…’) could be psychosocial stressors that impact the health of migrants.

## 6. Methodological outlook

A sustained discussion on the diversity of our society in the context of epidemiologic and public health research hinges on a continuous reflection of the concepts and categories applied, and on avoiding ascriptions-based generalisations. The concepts presented in this article are an important development of the conceptual basis of health monitoring at the RKI which allow an inclusion of several migration-sensitive components. The discussion of various migration-sensitive concepts and surveying instruments should provide a basis to better account for the heterogeneity and diversity of people in Germany in future data collection and analysis. Even though the large number of migration-related questions might increase the probability of non-response, and thus the generation of unusable data, it does provide more detailed insights into one’s history of migration (for example duration of stay, residency status or motives for migration). This should enable a better understanding of the concomitant health effects far beyond the indicator of migration background (yes/no) or of a generation within a family that has migrated. Factors such as the subjective belonging to a group and discrimination, religious creed as well as subjective social status may also be important factors to explain differences in health outcomes between people with and without a history of migration. This will allow for a more detailed description of these factors for example in the context of health survey data collection also with regard to possible interdependences and correlations, such as the connection between subjective social status and discrimination or a person’s feeling of belonging to the society. The concepts developed should potentially provide a basis to portray the health resources and burdens as well as the needs and requirements of diverse population groups in a more differentiated way. RKI health survey data could contribute to future discussions on equity in health and social participation as well as discrimination. Using the described concepts, the basis for further differentiated data analyses of the health of people with migration background can be improved, also enabling a responsible communication of the results to researchers, politics and the broader public. From our perspective, several concepts should not be used exclusively for people with a migration background but applied in perspective to all population groups, since some of them - such as discrimination or religion - impact the health of all people living in Germany.

When applying these concepts to improve the migration-sensitivity of health monitoring at the RKI and public health research in general, some methodological questions need to considered, which we discuss in the following. It will be important to create adequate framework conditions and adapt questionnaire instruments, measures that need to include new forms of reachability, discrimination-free settings and accessibility of surveys in different languages [35]. Using questionnaires in various languages requires a methodological discussion of the specific demands of questionnaire translation. Instead of a word-for-word translation, the aim will generally be for an idiomatic translation, i.e. one that clearly renders the meaning and concepts of a text in the foreign language. Within the IMIRA project, a standard operation procedure was developed that considers the team approach (also called committee approach) as an important element in the idiomatic translation of questionnaires. It intends to involve several native translators with different qualifications in the translation process to prevent translations from being both too vague and having too much of a personal style [72]. Finally, the quality of the instruments - with regard for example to validity - should also be proven for the translated versions. Cognitive pre-testing with participants from all relevant language groups including German should allow the identification of where improvements can be made.

Beyond translations, this applies to the entire research process. The participation of people with a migration background as well as of those who are affected by racist discrimination, yet who are not included in the migration background category according to the definition, would be essential to optimise research settings. This applies as much to the development and establishment of migration-sensitive concepts as to the application, evaluation and communication of the results. Furthermore, attention should be paid to discrimination-free framework settings for interviews, for example by training the intercultural and communicative capacities of interviewers [35].

In the future, beyond the four concepts presented here, it will be necessary to consider further aspects. These could include, for example, social resources and coping strategies, vigilance, resilience and self-efficacy. These approaches are part of a necessary shift of focus in the discourse on health and migration, which is too often focused on deficits, towards a capacities and resources oriented perspective.

Addressing the diversity of the population in the context of epidemiological analyses will require new approaches. Intersectional perspectives for example should, where possible, also be applied in epidemiology - social categories need to be analysed not separately but with regard to how they intersect each other. This emphasises the need for a discussion of how to apply and bring together a diverse set of analytical approaches in quantitative health research [73]. The analyses of different health settings may better reflect the situation of the population as a whole.

## Key statements

- Relevant migration-related characteristics such as language, sense of belonging and migration history should be surveyed without making generalising statements on degrees of ‘acculturation’.
- Surveying (antidiscrimination data provides the basis for a more differentiated analysis of the health of a heterogeneous population.
- Religion can influence health and serves as an important explanatory factor for health differences.
- Data on subjective social status can help analyse the effects of perceived social conditions and social mobility on the health of people with a migration history.
- In a longer term perspective, the concepts presented are to be applied not merely in surveys related to migration, but to all population groups.
